# Impact of Presumed Tree Nut and Peanut Allergy on Quality of Life at Different Ages

**DOI:** 10.3390/jcm12103472

**Published:** 2023-05-15

**Authors:** Maria Pasioti, Maria Savva, John Lakoumentas, Evangelia Kompoti, Michael Makris, Paraskevi Xepapadaki, Nikolaos G. Papadopoulos

**Affiliations:** 1 Allergy Department, 2nd Pediatric Clinic, National and Kapodistrian University of Athens, Athens General Children’s Hospital Panagioti and Aglaia Kyriakou, Thivon & Levadias, Ambelokipoi, 115 27 Athens, Greece; 2 Allergy Department, 2nd Pediatric Clinic, National and Kapodistrian University of Athens, Fidippidou Street, 4th Floor, Goudi, 115 27 Athens, Greece; 3 Allergology Department, “Laikon” General Hospital, 115 27 Athens, Greece; 4 Allergy Unit ‘D. Kalogeromitros’, 2nd Department of Dermatology and Venereology, Medical School, National and Kapodistrian University of Athens, University General Hospital “Attikon”, 124 62 Chaidari, Greece; 5 Division of Immunology, Immunity to Infection and Respiratory Medicine, Faculty of Biology, Medicine and Health, The University of Manchester, Manchester M13 9PL, UK

**Keywords:** tree nut allergy, peanut allergy, hazelnut allergy, pistachio allergy, cashew allergy, walnut allergy, almond allergy, quality of life

## Abstract

Tree nut and/or peanut allergy impairs patients’ quality of life, but data on the impact of age and the type of nut or peanut on the quality of life are lacking. To evaluate the impact at different ages, age-appropriate survey questionnaires accompanied by FAQLQ and FAIM were distributed to patients with suspected tree nut and/or peanut allergy who presented at the allergy departments of three hospitals in Athens. Out of 200 questionnaires distributed, 106 met the inclusion criteria (46 children, 26 teenagers, 34 adults). The median score of each age group for FAQLQ was 4.6 (3.3–5.1), 4.7 (3.9–5.5), and 3.9 (3.2–5.1) and for FAIM was 3.7 (3.0–4.0), 3.4 (2.8–4.0), and 3.2 (2.7–4.1), respectively. FAQLQ and FAIM scores were correlated with the reported probability of using the rescue anaphylaxis set upon reaction (15.4%, *p* = 0.04 and 17.8%, *p* = 0.02, respectively) and pistachio allergy (FAQLQ: 4.8 vs. 4.0, *p* = 0.04; FAIM: 3.5 vs. 3.2, *p* = 0.03). Patients with additional food allergies reported worse FAQLQ scores (4.6 vs. 3.8, *p* = 0.05). Worse FAIM scores were associated with younger age (−18.2%, *p* = 0.01) and the number of life-threatening allergic reactions (25.3%, *p* < 0.001). The overall impact of tree nut and/or peanut allergy on patients’ quality of life is moderate but differs with age, the type of nut, the use of adrenaline, and the number of previous reactions. The aspects of life affected and contributed factors also vary across age groups.

## 1. Introduction

Tree nut and/or peanut allergy (TNPA) is usually a lifelong, potentially life-threatening, disease [1] and represents one of the most common causes of food-induced hospital admissions and deaths [2]. Standard management includes strict avoidance of the culprit nut and rescue medication, mainly adrenaline, in case of anaphylaxis due to accidental exposure [3]. Oral immunotherapy has recently been approved as an alternative management of peanut allergy in children [4].

The worldwide prevalence of tree nut allergy is estimated between 0.05% and 4.9%. In Europe, the self-reported lifetime prevalence of peanut and tree nut allergy is 1.3% and 2.2%, respectively, with hazelnut being the most frequently implicated tree nut [1]. However, the prevalence of confirmed by oral food challenges TNPA is 0.2% and 0.5% [5].

Besides health issues, food allergy, similar to other chronic conditions, affects patients’ quality of life (QoL) [6,7]. The necessity of daily restrictions imposes a significant burden on patients’ daily lives, induces stress and anxiety, and subsequently affects their social life [8,9,10,11], especially in patients with peanut allergy [9]. A history of life-threatening and/or multiple previous allergic reactions, parental anxiety, and uncertainty about managing accidental reactions imposes an increased psychological burden [12,13,14]. Regarding tree nuts, data from a single study indicate a significant emotional and psychosocial burden in children [15].

Greece is a country with a low incidence of food allergy and equally low public awareness [16]. Although allergy services are provided by highly specialized practitioners in academic, public, and private settings, Greek adults reported worse levels of QoL than other European patients with food allergies [16,17]. There is no reported data regarding specifically the QoL of Greek patients with TNPA. In this context, we aimed to evaluate the impact of presumed TNPA (pTNPA) on QoL and possible associated factors.

## 2. Materials and Methods

To assess the QoL of patients with pTNPA, we utilized the Greek versions of the food allergy quality of life questionnaire for children aged 8–12 years old (FAQLQ-CF) [18], teenagers (FAQLQ-TF) [19], and adults (FAQLQ-AF) [20], with answers on a 7-point Likert scale (1 = no impairment, 2 = barely, 3 = a little bit, 4 = fairly, 5 = quite, 6 = very, 7 = extreme). The food allergy independent measure (FAIM), consisting of six questions on a 7-point Likert scale (1–7), was used as an independent measure based on patients’ expectations of food allergic outcomes upon accidental reactions due to their food allergy [21]. FAQLQ and FAIM questionnaires were self-completed by patients of every age.

FAQLQ-CF included the following domains: allergen avoidance (AA), dietary restrictions (DR), risk of accidental exposure (RAE), and emotional impact (EI). FAQLQ-TF included three domains: allergen avoidance and dietary restrictions (AADR), RAE, and EI, while FAQLQ-AF had four domains: AADR, EI, RAE, and food allergy related health (FAH).

Participants’ demographic information and allergy history were collected through three standardized age-appropriate survey questionnaires. These questionnaires were completed by teenage and adult patients and the parents of children aged 8–12 years old.

To clinically interpret the questionnaires’ results, we classified scores <4 as minor clinical importance, whereas scores ranging from 4 to 7 indicated major clinical importance. FAIM, FAQLQ total, and specific domain scores were investigated for correlations with factors assessed in the survey questionnaire.

Eligible for participation were Greek-speaking patients of both sexes who visited the Allergy Department of the 2nd Pediatric Clinic of National and Kapodistrian University of Athens, “Panagiotis and Aglaia Kyriakou” Children’s Hospital, and the collaborating centers of the allergology department of “Laiko” General Hospital and the allergy unit “D. Kalogeromitros”, 2nd Department of Dermatology and Venereology of National and Kapodistrian University of Athens, Attikon Hospital, all located in Athens, between June 2018 and February 2020. All patients who reported avoiding at least one tree nut (walnut, pistachio, cashew, hazelnut, almond) or peanut because of an assumed IgE-mediated allergy were invited to participate after receiving oral and written information indicating the purpose of the study. Participation was anonymous and voluntary.

## 3. Statistical Analysis

All numerical answers and outcomes based on the given questionnaires were considered non-parametric continuous variables in the statistical analysis. Non-normality was validated with the Shapiro–Wilk test for composite normality. Descriptive statistics were given in the form of median (Q1–Q3) for the continuous variables and as count (%) for the discrete ones. To assess differences across age groups, Pearson’s chi-squared test of independence was performed for qualitative variables and the Kruskal–Wallis test for quantitative variables. Pairwise non-parametric correlations of scale variables were estimated with the Kendall’s tau correlation estimator. The 30% and 50% cut-offs were used to characterize a correlation estimate as low, moderate, or high. Associations between the Likert-scaled variables and discrete factors were examined with Wilcoxon’s rank sum test.

All statistical tests were taken as two-sided and statistical significance was considered at the level of 5%. Missing values were not imputed. The statistical analysis was held in R and RStudio, both of which are open-source products.

Separate analyses were conducted to include patients with suspected allergy only to a single nut. Using the Mann–Whitney test, we compared these patients’ total FAQLQ and FAIM scores.

To estimate correlations between the type of nut and the QoL of patients with pTNPA, cashew and pistachio were considered as one nut. In clinical practice, patients with allergy to cashew or pistachio are advised to avoid both nuts because of their common biological heritage (Anacardiaceae family) [22,23]. Regarding the walnut family, we only addressed walnut allergy, as other members of the walnut family such as pecan are uncommon in Greek culinary habits. However, as part of our questionnaire, participants were asked to also respond to an open question regarding allergies to other nuts; none of the patients were allergic to pecan.

Surveys missing more than 20% of answers per questionnaire were excluded from the analysis.

## 4. Results

### 4.1. Characteristics of the Study Participants

In total, from the 127 questionnaires distributed, 121 were obtained, of which 106 (46 from children, 26 from teenagers, and 34 from adults) were included in this analysis (Supplementary Figure S1). In the total cohort, the median patient age was 14 years old (CI: 10–18) and 69 were males (65.7%). Most patients reported either allergy to pistachio and cashew: *n* = 67 (64.4%) and *n* = 64 (61.5%), respectively, or previous reactions: pistachio: 39 participants (42.9%) and cashew: 36 (39.6%). Descriptive data, including demographic, personal history of allergy-associated diseases, and information regarding reaction to all tree nuts, are depicted in Table 1.

### 4.2. FAQLQ and FAIM Scores

The median FAQLQ and FAIM scores of the total cohort and by age group are shown in Table 2 and Supplementary Figure S1.

### 4.3. Factors Correlating with QoL

In the total sample, older age was associated with slightly better (lower) FAIM scores (−18.2%, *p* = 0.01). Patients with additional food allergies presented worse FAQLQ scores than those with only pTNPA (4.6 vs. 3.8, respectively, *p* = 0.05). FAQLQ and FAIM scores were significantly correlated with the reported probability of using the rescue anaphylaxis set upon reaction (15.4%, *p* = 0.04 and 17.8%, *p* = 0.02, respectively). The FAIM score also positively correlated to the number of life-threatening allergic reactions reported (25.3%, *p* < 0.001).

Patients allergic to pistachio had worse (higher) FAQLQ and FAIM scores than patients whose nut panel did not include pistachio (FAQLQ: 4.8 vs. 4.0, *p* = 0.04; FAIM: 3.5 vs. 3.2, *p* = 0.03). The total FAQLQ and FAIM scores by age group and type of allergen are demonstrated in Supplementary Table S1. A subgroup analysis, including participants who reported allergy only to a single nut, showed that assumed pistachio or cashew allergy was associated with worse FAQLQ (5.0 vs. 3.0, respectively, *p* < 0.01) and FAIM (3.8 vs. 2.8, respectively, *p* < 0.01) scores. On the contrary, patients with suspected walnut allergy had better FAQLQ (2.7 vs. 4.8, *p* = 0.02) and FAIM (2.5 vs. 3.7, *p* = 0.01) scores than patients with other pTNPA (Supplementary Table S2).

Regarding children, presumed allergy to walnut, among other nuts, was associated with better (lower) overall FAQLQ scores than those allergic to nuts other than walnut (4.2 vs. 4.9, *p* = 0.02), better risk of accidental exposure scores (3.5 vs. 4.6, *p* = 0.01), and better emotional impact scores (4.3 vs. 6.2, *p* < 0.01). Children with previous allergic reactions to walnut also scored lower in the domain of emotional impact (4.1 vs. 6.0, *p* < 0.01) and the overall FAQLQ score (3.6 vs. 4.9, *p* = 0.03) compared with children who reported reactions to any tree nut and/or peanut other than walnut.

Higher dietary restrictions scores were associated with the probability of using an anaphylactic set (24.0%, *p* = 0.04). The number of nuts the child was allergic to negatively and moderately correlated with the risk of accidental exposure score (−22.4%, *p* = 0.05). Notably, the pediatric FAIM score was correlated with the number of life-threatening anaphylactic experiences (42.4%, *p* < 0.01) and the times when an adrenaline injection was administered (53.9%, *p* = 0.05). The number of life-threatening experiences was positively related to higher emotional impact scores in children (28.7%, *p* = 0.05).

In poly-nut allergic teenagers, suspected allergy to pistachio or almond correlated with worse FAQLQ scores (5.0 vs. 3.4, *p* = 0.05 and 5.5 vs. 4.5 *p* < 0.01, respectively) and worse allergen avoidance and dietary restrictions scores (5.2 vs. 2.9, *p* = 0.02 and 5.8 vs. 4.8, *p* = 0.01, respectively). Patients with co-allergy to almond among other nuts had worse FAIM, risk of accidental exposure, and emotional impact scores (4.0 vs. 3.2, *p* = 0.04, 5.2 vs. 4.1, *p* = 0.01, and 5.6 vs. 4.3, *p* = 0.03, respectively).

The presence of additional food allergies in teenagers was associated with worse (higher) FAQLQ (5.4 vs. 4.4, *p* = 0.03), allergen avoidance and dietary restrictions (5.6 vs. 4.3, *p* = 0.01), and risk of accidental exposure scores (5.0 vs. 4.0, *p* = 0.04).

The FAIM scores were also strongly and positively correlated with the number of life-threatening experiences reported (52.3%, *p* = 0.01).

In adults, females had worse (higher) scores in FAQLQ (4.6 vs. 3.0, *p* = 0.01), risk of accidental exposure (4.1 vs. 2.8, *p* = 0.02), emotional impact (4.3 vs. 3.1, *p* < 0.01), food allergy related health (4.8 vs. 3.3, *p* < 0.01), and FAIM (3.5 vs. 2.7, *p* = 0.03) than males.

Additionally, in adults, greater availability of the treatment set and greater probability of using it were correlated with worse scores in FAQLQ (49.8%, *p* = 0.001 and 37.5%, *p* < 0.01), allergen avoidance and dietary restrictions (52.4%, *p* < 0.001 and 42.9%, *p* < 0.01), risk of accidental exposure (47.1%, *p* < 0.01 and 34.5%, *p* = 0.02), food allergy related health (31.5%, *p* = 0.04 and 30.0%, *p* = 0.04), and FAIM (30.3%, *p* = 0.05 and 37.1%, *p* = 0.01), while worse emotional impact scores correlated with greater availability of treatment set only (36.7%, *p* = 0.02).

Adults who reported previous use of adrenaline had better (lower) food allergy related health scores compared with those who had never used adrenaline before (2.3 vs. 4.3, *p* = 0.04). Furthermore, the adult participants with additional food allergies had worse scores in FAQLQ (4.3 vs. 2.9, *p* = 0.04) and allergen avoidance and dietary restrictions (4.9 vs. 3.0, respectively, *p* = 0.02) than those with pTNPA only. Finally, adults who reported allergy to cashew, among other nuts, had better food allergy related health scores than those who were allergic to nuts other than cashew (3.3 vs. 4.5, *p* = 0.05).

## 5. Discussion

To our knowledge, this is the first study assessing the effect of suspected peanut and tree nut allergy on the quality of life across age groups. Our results show that pTNPA moderately affects the QoL of life of patients, with teenagers reporting the worst outcomes and adults the best. In children, the most affected domain is the emotional impact (EI), indicating a fear of accidental reactions and frustration with their allergy. This finding should be a concern to both healthcare and education professionals, regarding the way they handle and communicate the potential impact and danger of TNPA to children causing a greater emotional burden. In teenagers and adults, QoL is mainly affected by the constant vigilance that is essential for safe food choices and the limitations in social life, as reflected in allergen avoidance and dietary restrictions (AADR). Adolescence is a stage of life where teenagers start to take responsibility for their allergy management and develop their own social life. Therefore, the intense frustration they experience because of the daily restrictions could be responsible for the greater impact on their QoL.

The correlation between QoL, the availability of an anaphylaxis rescue set, and the probability of using it when in need has not been reported before. Patients with an increased QoL burden and those who perceived their allergy as more severe reported a higher likelihood of using their anaphylaxis set upon reaction. That finding was more pronounced in adults and consistent in all domains of FAQLQ. It is plausible that patients who have experienced life-threatening reactions consider themselves to be suffering from severe allergy and expect to experience a threatening outcome upon accidental exposure (higher FAIM and EI scores). These patients report difficulties in complying with allergen avoidance (higher RAE score), which affects their food choices and social activities (higher AADR score) and renders them anxious about their wellbeing (higher FAH score). Of course, reverse causation, e.g., the need to have on hand the anaphylaxis set all the time might impact the FAQLQ scores, cannot be excluded. Nevertheless, Cummings et al. [15] did not find any differences in QoL regarding whether the child carried the prescribed auto-injector or not.

Another novel finding is the correlation of specific tree nuts with QoL. Presumed allergy to pistachio and almond was found to impose a significant burden on patients’ QoL and their perception of the severity of their allergy. This effect was more profound in teenagers. Reported walnut allergy was associated with better QoL scores, especially in children. In the adult population, assumed cashew allergy was correlated with better scores in the food allergy related health domain. The effect of food allergies, in general, and TNPA on patients’ QoL could vary substantially between countries with different culinary customs, suggesting that results are better interpreted in the specific geographic and cultural context [17,24].

Pistachio and cashew nuts are known to cause severe allergic reactions, even in traces [25,26]. Pistachio is widely used in Greek pastry and is a typical snack; it is also used in many prepacked foods. Consequently, an allergy to Anacardiaceae usually manifests as an allergy to pistachio and patients are thereafter advised to avoid cashew and pistachio. The difficulties in complying with pistachio avoidance are reflected in the allergy avoidance and dietary restriction (AADR) scores. On the other hand, cashew is not typically used in the Greek diet; ready-to-eat sauces, prepacked, and restaurant meals are the primary cause of accidental ingestion, the avoidance of which does not negatively affect the patient’s overall health. Almond, although seldomly causes severe allergic reactions [27], is widely used in the Greek diet. Regarding walnut allergy, many Greek patients report symptoms in the context of LTP syndrome, where traces usually do not cause reactions [28]. The milder clinical manifestations of LTP syndrome, compared with the severe anaphylaxis episodes caused by pistachio and/or cashew accidental consumption, reflect the different perception in the FAQLQ scores. Although walnut represents a common ingredient in desserts, it is infrequently used in standardized products and traces for pediatric consumption, facilitating its avoidance.

In our study population, younger patients perceived their allergy more frequently as severe. This finding has been reported before [15,29] and might reflect the fact that older patients are familiar with coping with their allergies.

In the adult population, females reported significantly greater impairment in almost every aspect of QoL than males. This was in line with previous studies [30], showing that the burden of food allergy more greatly affects the female population. This was not the case in children and adolescents, even though other studies have reported sex and gender differences in QoL of younger patients with food allergy [15,30,31,32], possibly because of the male dominance in our pediatric and adolescent population. The impact of sex in allergic diseases has been reported before and has been attributed to the influence of sex steroids on immune responses [33,34]. Additionally, females may experience worse QoL because of gender differences in coping strategies, risk evaluation, and role expectations compared to males [35].

In children, the reverse association between the number of tree nuts and the risk of accidental exposure (RAE) scores indicates that the more tree nuts the child is allergic to the less the child is affected by the risk of accidental exposure. Although this finding seems contradictory, children allergic to more nuts are usually advised to avoid all nuts and traces, minimizing the risk of accidental exposure.

We did not find any association between the number or the severity of reactions (assessed by adrenaline use or the experience of a life-threatening reaction) and the FAQLQ scores, in line with some previous studies on food allergy [30,31,36] or TNPA [9,15]. The experience of a life-threatening reaction correlated with FAIM scores, as expected. The number of reactions treated with adrenaline was also correlated with the FAIM score in children. On the other hand, adults who had ever used adrenaline to treat a reaction reported better FAH scores than those who had never used adrenaline. That finding can indicate that the experience of self-administered adrenaline confers the patients with the confidence of being capable of treating an allergic reaction. Nevertheless, Acaster et al. [12] reported that parents of children with peanut allergy stated a higher burden on their child’s life depending on the number and severity of reactions, while Pinczower et al. [10] reported that experiencing anaphylaxis deteriorated the QoL of children with food allergies. In our population, there were few reactions reported. We might have failed to show an association because there were few reports about life-threatening reactions and adrenaline use in our population. On the other hand, it is possible that, in tree nut and peanut allergy, the constant vigilance required to avoid reactions has a more considerable effect on patients’ QoL than the reactions themselves.

In our population, the prescription of an adrenaline auto-injector did not affect patients’ QoL. Previous studies [10,15,37] have reported conflicting results regarding the prescription of auto-injectors and the QoL of patients. Cummings et al. [15] found that the prescription of an auto-injector improved the QoL of mothers but had no effect on the QoL of their children. On the other hand, Pinczower et al. [10] and, more recently, Warrent et al. [37] found worse QoL in patients with food allergies being prescribed adrenaline. Finally, Saleh-Langenberg et al. [30] did not find any association between the QoL of patients with food allergy and being prescribed an auto-injector.

There are certain limitations in our study. The questionnaires used (FAQLQ.FAIM) were not explicitly designed for TNPA, while the Greek translation has only been validated for the pediatric and adult forms [17,38]. Nevertheless, these questionnaires have been used widely for peanut and tree nut allergy [39,40,41,42,43,44,45,46], making results comparable. Although not officially validated in Greek, the teenage forms consisted of similar vocabulary as the pediatric and adult forms and participants did not report any comprehension difficulties.

Another potential limitation of this study was the relatively small sample size for the subgroup analysis conducted based on the single type of tree nut and/or peanut the participants were allergic to, as most of the patients reported multiple pTNPA.

Participants in this study were recruited among patients visiting an allergy unit for reported TNPA, whose allergy was not necessarily objectively diagnosed by a healthcare professional. Therefore, we cannot exclude the possibility that some of them falsely presumed that they, or their children, suffered from TNPA. Nevertheless, the existing literature suggests that the impact of food allergy does not seem to differ between children whose parents perceive that they have food allergy and those who are confirmed as allergic to a particular food [47], hence we believe that our results do reflect the real burden of TNPA on QoL. The same stands for information regarding allergic reactions, treatment required, and allergic comorbidities. As this was an anonymous and self-reported survey, validation of the data was not possible. On the other hand, patients attending specialized centers for food allergy tend to report better QoL than unselected patients with the same diagnosis [46]. Finally, we cannot rule out the possibility that some of the burden reported was due to other food allergies, since the FAQLQ.FAIM scores were significantly worse in patients with co-existing food allergies.

This is the first study on the impact of pTNPA on QoL to include participants of all ages, with self-reported data even from children, investigating possible associated factors, including the type of tree nut. Our findings on pTNPA’s burden on patients’ QoL are similar to the impact of peanut allergy, as reported by Acaster et al. [12], and allergy to staple foods (cows’ milk, hens’ eggs, and wheat), as reported by Protudjer et al. [48].

Although the impact of current diagnostic procedures, such as the oral prοvocational tests, has not been within the scope of our study, we assume that they may also affect patients’ QoL. A recent systematic review by Kansen et al. [49] suggests that oral food challenge is associated with improved QoL of patients with food allergy and reduced parental burden. The present strategies of TNPA management could benefit from the results of our study. As there is currently no cure for food allergy, oral immunotherapy focuses on increasing the threshold and decreasing the severity of accidental reactions, which may presumably improve patients’ lives. Unveiling the factors that contribute to patient wellbeing helps to better recognize those who will benefit best from the new approaches.

## 6. Conclusions

Albeit tree nut and peanut allergy can lead to potentially life-threatening allergic reactions, its overall impact on the quality of life of patients is moderate, with teenagers and children affected more than adults. The domains affected vary according to different ages and by the nuts to which the patient is allergic. The finding that presumed pistachio and almond (which are widely used in Greece) allergies are associated with worse QoL, indicates that the surveillance required to avoid reaction may affect patients more than the reactions themselves. To support this, the experience of a life-threatening reaction affected patients’ perception of the severity of the allergy and no other domains of their life. Gender seems to play an additional role, with women affected more than men, at least in adult life. As there is currently no cure for food allergy, unveiling the factors that contribute to patient wellbeing helps in the design of better management approaches.

## Figures and Tables

**Table 1:** Descriptive statistics of the analyzed variables in the form of median (Q1–Q3) for quantitative variables and count (%) per level for qualitative variables for children, teens, adults, and all.

Variable	Children (*n* = 46)	Teens (*n* = 26)	Adults (*n* = 34)	* *p*-Value	All (*n* = 106)
**Male Sex**	33 (72%)	21 (81%)	15 (45%)	**<0.01**	69 (66%)
Missing Values	0	0	1		1
**Age (years)**	10 (9–11)	15 (14–16)	24 (20–33)	na	14 (10–18)
Missing Values **	3	4	6		13
**Presumed Tree Nut and/or Peanut Allergy**					
Walnut	24 (53%)	14 (54%)	22 (67%)	0.45	60 (58%)
Pistachio	34 (76%)	22 (85%)	11 (33%)	**<0.001**	67 (64%)
Cashew	35 (78%)	20 (77%)	9 (27%)	**<0.001**	64 (62%)
Hazelnut	23 (51%)	13 (50%)	14 (42%)	0.73	50 (48%)
Almond	14 (31%)	8 (31%)	12 (36%)	0.86	34 (33%)
Peanut	25 (56%)	16 (62%)	19 (58%)	0.89	60 (58%)
Missing Values	1	0	1		2
**Consuming Other Nuts**				0.36	
Never	18 (39%)	9 (35%)	7 (21%)		34 (32%)
Yes, without Restrictions	13 (28%)	6 (23%)	14 (41%)		33 (31%)
Sometimes, with Restrictions	15 (33%)	11 (42%)	13 (38%)		39 (37%)
Missing Values	0	0	0		0
**Total No. of TNP with co-Allergy** (Cashew/Pistachio = 1)	3 (1–4)	3 (1–4)	2 (1–4)	0.69	3 (1–4)
Missing Values	1	0	1		2
**Age of 1st TNP Reaction (years)**				**<0.001**	
<2	9 (20%)	8 (32%)	2 (7%)		19 (19%)
2–5	25 (57%)	10 (40%)	4 (13%)		39 (39%)
6–12	5 (11%)	4 (16%)	9 (30%)		18 (18%)
13–18	na	0 (0%)	11 (37%)		11 (11%)
>18	na	na	0 (0%)		0 (0%)
Never Had a Reaction	5 (11%)	3 (12%)	4 (13%)		12 (12%)
Missing Values	2	0	1		3
**TNP of the 1st Reaction**				**0.001**	
Walnut	5 (13%)	0 (0%)	9 (33%)		14 (16%)
Pistachio	6 (15%)	9 (39%)	1 (4%)		16 (18%)
Cashew	9 (23%)	3 (13%)	3 (11%)		15 (17%)
Hazelnut	11 (28%)	2 (9%)	3 (11%)		16 (18%)
Almond	0 (0%)	2 (9%)	4 (15%)		6 (7%)
Peanut	6 (15%)	5 (22%)	2 (7%)		13 (15%)
Other	2 (5%)	2 (9%)	5 (19%)		9 (10%)
Missing Values	6	3	6		15
**TNP with Allergic Reactions**					
Walnut	10 (26%)	6 (25%)	16 (55%)	**0.02**	32 (35%)
Pistachio	15 (40%)	16 (67%)	8 (28%)	**0.01**	39 (43%)
Cashew	17 (45%)	13 (54%)	6 (21%)	**0.03**	36 (40%)
Hazelnut	10 (26%)	7 (29%)	10 (35%)	0.77	27 (30%)
Almond	2 (5%)	6 (25%)	11 (38%)	**<0.01**	19 (21%)
Peanut	7 (18%)	13 (54%)	13 (45%)	**<0.01**	33 (36%)
Missing Values	8	2	5		15
**Total No. of Reactions**	2 (1–3)	1 (1–3)	2 (1–3)	0.79	2 (1–3)
Missing Values	2	5	6		13
No. of Reactions Last Year	0 (0–0)	0 (0–1)	0 (0–1)	0.53	0 (0–1)
Missing Values	15	8	10		33
No. of Life-Threatening Reactions	0 (0–1)	0 (0–1)	1 (0–1)	0.82	0 (0–1)
Missing Values	14	9	13		36
**Adrenaline Prescription**	35 (81%)	19 (83%)	13 (50%)	**<0.01**	67 (73%)
Missing Values	3	3	8		14
**Anaphylaxis Set Availability** (0: Never–6: Always)	5 (3–5)	3 (2–5)	5 (3–6)	**0.04**	4 (3–5)
Missing Values	3	3	7		13
**Anaphylaxis Set Use Probability** (0: Never–6: Always)	5 (3–5)	5 (4–6)	4 (2–5)	0.55	5 (3–5)
Missing Values	3	3	7		13
**Adrenaline Use**					
Ever	6 (13%)	10 (40%)	7 (23%)	**0.04**	23 (23%)
Missing Values	16	7	10		33
No. of Reactions	1 (0–1)	1 (1–1)	1 (1–2)	0.20	1 (1–1)
Missing Values	35	17	27		79
No. of Reactions Last Year	0 (0–0)	0 (0–1)	0 (0–0)	0.11	0 (0–0)
Missing Values	36	18	26		80
**Other Food Allergy**	20 (44%)	16 (62%)	20 (61%)	0.20	56 (53%)
Missing Values	0	0	1		1
**Allergic Rhinitis**	17 (39%)	9 (38%)	19 (61%)	0.10	45 (46%)
Missing Values	0	0	2		2
**Allergic Conjunctivitis**	6 (13%)	5 (22%)	6 (19%)	0.64	17 (17%)
Missing Values	0	0	2		2
**Atopic Dermatitis**	22 (48%)	6 (26%)	10 (32%)	0.16	38 (38%)
Missing Values	0	1	2		3
**Drug Allergy**	2 (5%)	5 (23%)	4 (13%)	0.11	11 (12%)
Missing Values	1	0	0		1
**Venom Allergy**	1 (3%)	1 (5%)	1 (3%)	0.91	3 (4%)
Missing Values	0	0	0		0
**Allergic Asthma**	12 (26%)	9 (36%)	7 (20%)	0.41	28 (27%)
Missing Values	0	0	0		0

* Differences across age groups were examined with Pearson’s chi-squared test of independence for qualitative variables and the Kruskal–Wallis test for quantitative variables. ** In cases of missing values regarding participants’ age, questionnaires were distributed according to their age group. TNP—tree nut and/or peanut. na—non-applicable.

**Table 2:** Descriptive statistics of the available questionnaire averages in the form of median (Q1–Q3) for children, teens, adults, and all.

	Children (*n* = 46)	Teenagers (*n* = 26)	Adults (*n* = 34)	All (*n* = 106)	* *p*-Value
**FAQLQ Score**	4.6 (3.3–5.1)	4.7 (3.9–5.5)	3.9 (2.8–4.9)	4.5 (3.2–5.1)	0.08
Allergen Avoidance Score and Dietary Restrictions Score	3.7 (3–5.1) 4.3 (2.9–5)	5.0 (3.8–5.8)	4.2 (2.6–5.4)	na	
Risk of Accidental Exposure	4.1 (2.8–5.2)	4.4 (3.5–5.1)	3.6 (2.3–5.1)	na	
Emotional Impact	5.0 (4.0–6.2)	4.6 (4.1–5.9)	3.7 (3.2–5)	na	
Food Allergy Related Health	na	na	4.2 (2.8–5.3)	na	
**FAIM Score**	3.7 (3.0–4.0)	3.4 (2.8–4.0)	3.2 (2.7–4.1)	3.4 (2.8–4.0)	0.44

**FAQLQ**—food allergy quality of life questionnaire. **FAIM**—food allergy independent measure. * Kruskal–Wallis test was performed to indicate differences across age groups. na—non-applicable.

## Data Availability

Data are available on request due to restrictions.

## Supplementary Materials

The following supporting information can be downloaded at: https://www.mdpi.com/article/10.3390/jcm12103472/s1.

## Abbreviations

TNPA	Tree nut and/or peanut allergy
pTNPA	Presumed tree nut and/or peanut allergy
QoL	Quality of life
CF	Child form
TF	Teenager form
AF	Adult form
FAQLQ	Food allergy quality of life questionnaire
FAIM	Food allergy independent measures
AA	Allergen avoidance (AA)
DR	Dietary restrictions
AADR	Allergen avoidance and dietary restrictions
RAE	Risk of accidental exposure
EI	Emotional impact
FAH	Food allergy related health
