# Supplementary material for: Impact of Presumed Tree Nut and Peanut Allergy on Quality of Life at Different Ages
Source: J Clin Med. 2023 May 15;12(10):3472. doi: 10.3390/jcm12103472 (PMC10218855; doi:10.3390/jcm12103472)
Supplement: Supplementary file 1 [file jcm-12-03472-s001.zip › jcm-2202803-supplementary.pdf]

Supplementary Materials

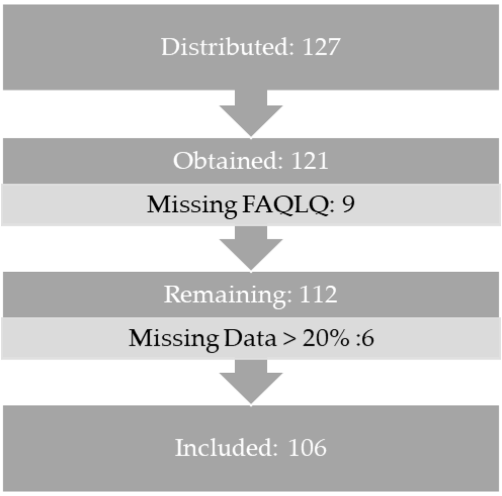

Supplementary Figure S1. Questionnaires included in the study

Supplementary Table S1. Total-FAQLQ and FAIM scores by age-group and type of allergen

TOTAL FAQLQ SCORE

	ALL	CHILDREN	TEENAGERS	ADULTS
**Walnut Allergy**	4.2 (2.9 – 5.0)	4.2 (2.7 - 4.9)	5.0 (3.4 - 5.5)	3.9 (2.7 - 4.7)
**Pistachio Allergy**	4.8 (3.6 - 5.2)	4.6 (3.3 – 5.0)	5.0 (4.4 - 5.6)	4.8 (3.8 - 5.1)
**Cashew Allergy**	4.6 (3.3 - 5.1)	4.7 (3.3 – 5.0)	4.7 (4.0 - 5.3)	4.0 (1.8 – 5.0)
**Hazelnut Allergy**	4.2 (3.0 - 5.1)	4.2 (3.0 - 4.9)	5.1 (4.1 - 5.5)	3.8 (2.7 - 4.2)
**Almond Allergy**	4.3 (3.4 - 5.4)	4.2 (3.3 - 4.7)	5.5 (5.2 - 6.1)	4.0 (3.0 - 4.8)
**Peanut Allergy**	4.6 (3.7 - 5.4)	4.7 (3.7 - 5.1)	4.8 (4.3 - 5.5)	4.3 (3.0 – 5.0)

FAIM SCORE

	ALL	CHILDREN	TEENAGERS	ADULTS
**Walnut Allergy**	3.3 (2.7 – 4.0)	3.3 (2.6 – 4.0)	3.2 (2.9 - 4.0)	3.2 (2.5 - 3.8)
**Pistachio Allergy**	3.5 (3.1 - 4.1)	3.7 (3.2 – 4.0)	3.5 (3.0 – 4.0)	3.3 (2.8 – 5.0)
**Cashew Allergy**	3.5 (3.0 – 4.0)	3.7 (3.2 – 4.0)	3.3 (2.8 - 3.9)	3.5 (2.7 - 3.8)
**Hazelnut Allergy**	3.3 (2.7 – 4.0)	3.3 (2.7 - 3.9)	3.7 (3.0 - 5.2)	3.3 (2.7 - 4.1)
**Almond Allergy**	3.7 (2.9 - 4.5)	3.8 (2.8 – 4.0)	3.9 (3.4 - 5.2)	3.3 (2.8 - 4.3)
**Peanut Allergy**	3.5 (2.8 - 4.0)	3.8 (3.0 – 4.0)	3.3 (2.8 - 4.0)	3.5 (2.7 - 4.4)

Supplementary Table S2. FAIM and Total-FAQLQ scores for patients with presumed allergy only to a single nut *(Not enough data for allergy only to almond or hazelnut for this analysis)*

	Walnut Allergy only			***Cashew/Pistachio Allergy only***			***Peanut Allergy only***		
	YES	*NO*	*p-value*	*YES*	*NO*	*p-value*	*YES*	*NO*	*p-value*
FAIM Score	2.5	3.7	**0.01**	3.8	2.8	**<0.01**	*3.5*	*3.3*	*0.95*
Total-FAQLQ Score	2.7	4.8	**0.02**	5.0	3.0	**<0.01**	*4.7*	*4.0*	*0.73*
